# Supplementary material for: Tailoring Nanoadsorbent Surfaces: Separation of Rare Earths and Late Transition Metals in Recycling of Magnet Materials
Source: Nanomaterials (Basel). 2022 Mar 16;12(6):974. doi: 10.3390/nano12060974 (PMC8950031; doi:10.3390/nano12060974)
Supplement: Supplementary file 1 [file nanomaterials-12-00974-s001.zip › nanomaterials-1611847-supplementary.pdf]

nanomaterials

Supplementary Information

Tailoring Nanoadsorbent Surfaces: Separation of Rare Earths and Late Transition Metals in Recycling of Magnet Materials

Ani Vardanyan [1,*], Anna Guillon [1], Tetyana Budnyak [2] and Gulaim A. Seisenbaeva [1,*]

[1] Department of Molecular Sciences, Swedish University of Agricultural Sciences, P.O. Box 7015, 75007 Uppsala, Sweden; anna.guillon@slu.se

[2] Department of Materials Science and Engineering, Division of Nanotechnology and Functional Materials, Uppsala University, P.O. Box 35, 75103 Uppsala, Sweden; tetyana.budnyak@angstrom.uu.se

* Correspondence: ani.vardanyan@slu.se (A.V.); gulaim.seisenbaeva@slu.se (G.A.S.)

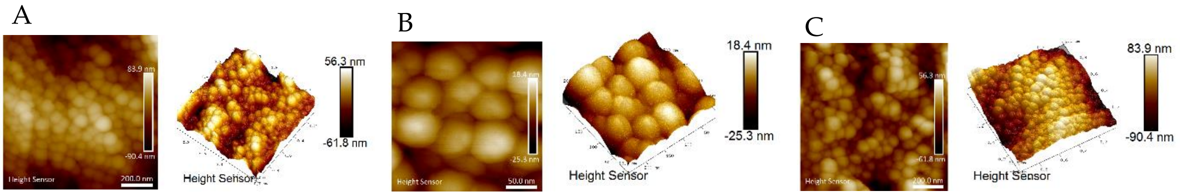

Figure S1. AFM images for acid treated SiO_2 NPs functionalized with **L5** (**A**,**B**) and **L3** ligands (**C**).

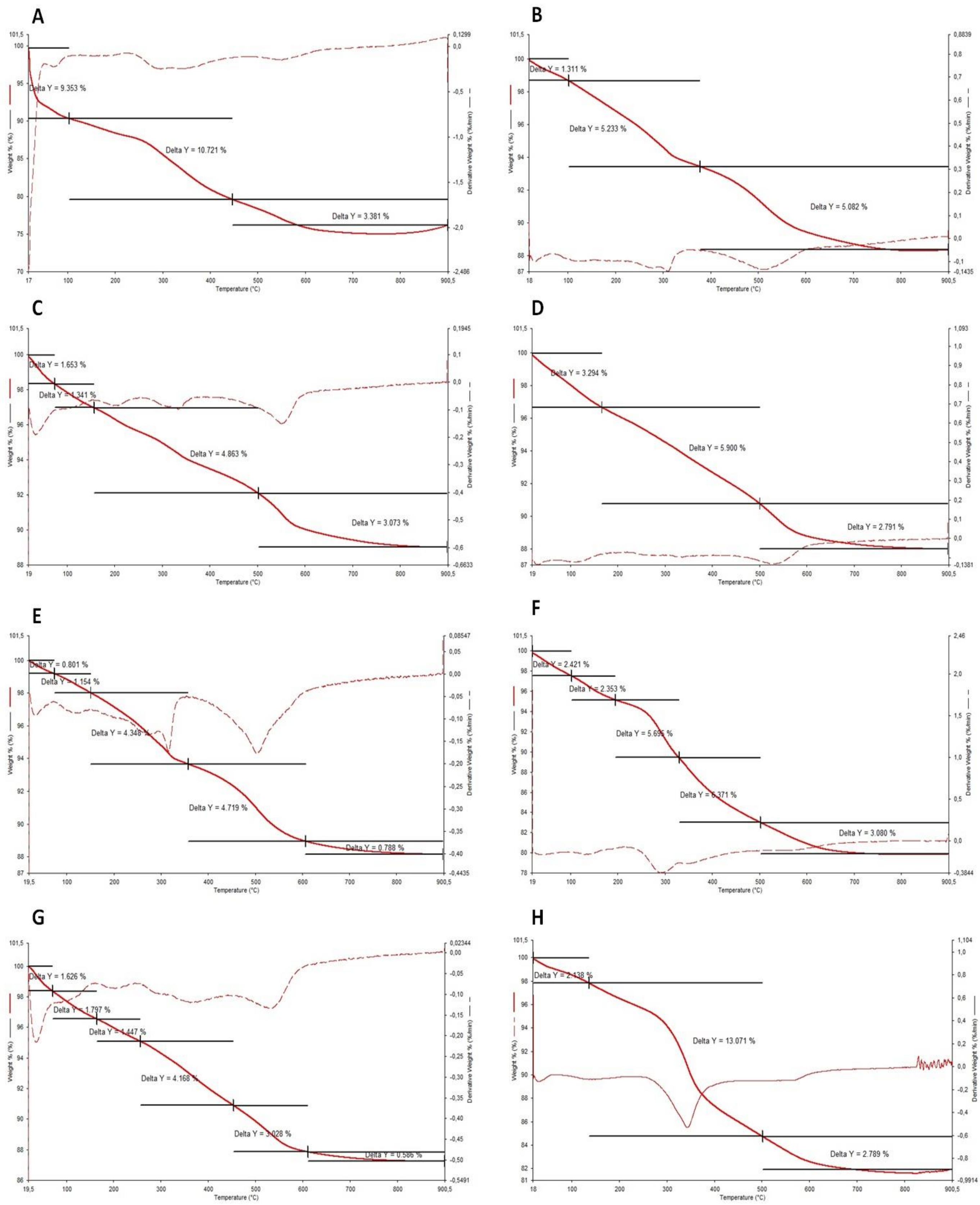

Figure S2. TGA analyses of grafted SiO_2 NPs: (**A**)-**L1**, (**B**)-**L2**, (**C**)-**L3**, (**D**)-**L4**, (**E**)-**L5**, (**F**)-**L3_acid**, (**G**)-**L4_acid**, (**H**)-**L5_acid**.

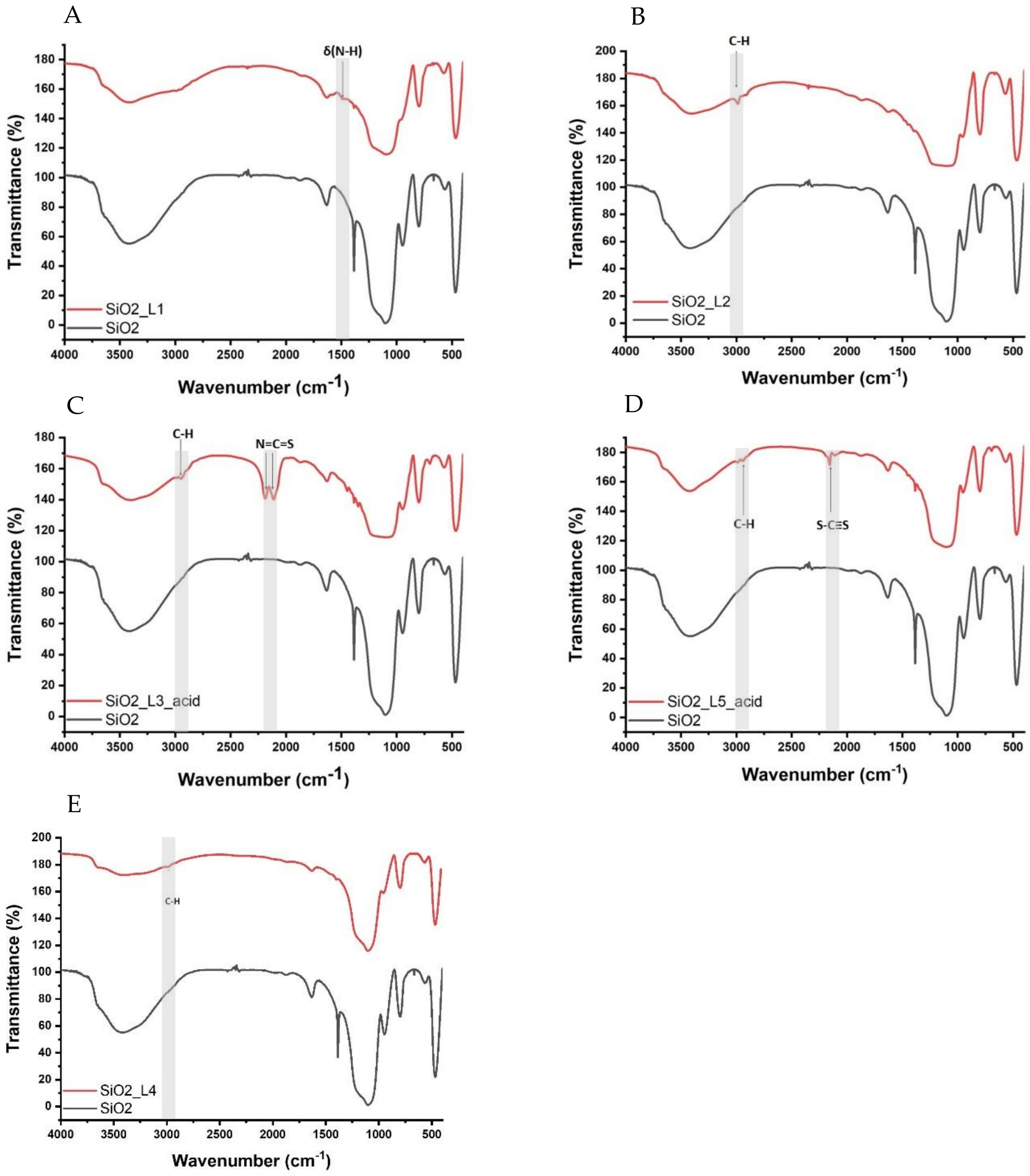

Figure S3. FTIR spectra of synthesized nanoparticles grafted with (**A**)-**L1,** (**B**)-**L2**, (**C**)-**L3_acid**, (**D**)-**L5_acid**, (**E**)-**L4**.

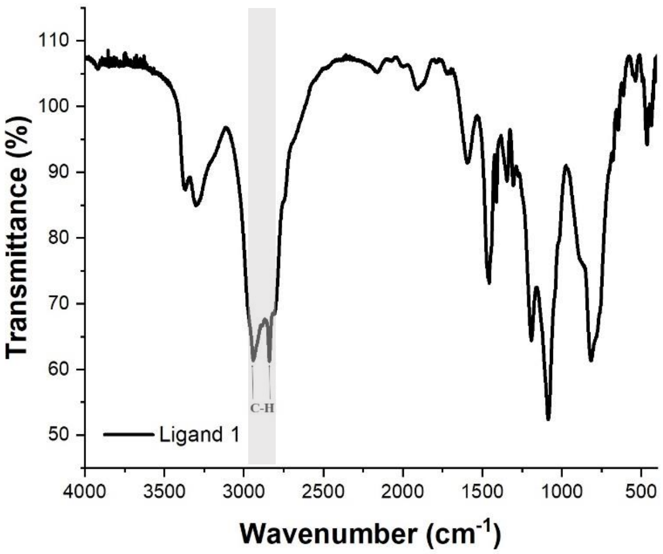

Figure S4. FTIR spectra of pure ligand (Ligand 1) before grafting.

Table S1. List of parameters obtained from Langmuir and Freundlich linear isotherm models.

**Model**		**L1**	**L2**	**L3**	**L1**	**L2**	**L3**	**L1**	**L2**	**L3**	**L1**	**L2**	**L3**
			**Co^{2+}**			**Ni^{2+}**			**Sm^{3+}**			**Nd^{3+}**	
**Langmuir**	**q_m**	1.90	0.86	0,83	1.82	0.62	0.60	1.31	0.64	0.78	1.12	0.6	0.86
	**K_L**	0.35	0.14	0.38	0.48	0.37	0.88	0.27	0.36	0.27	0.16	0.26	0.16
	**R^2**	0.99	0.98	0.99	0.98	0.99	0.99	0.99	0.99	0.99	0.99	0.99	0.99
**Freundlich**	**K_f**	0.43	0.09	0,19	0.47	0.13	0.25	0.23	0.16	0.17	0.14	0.12	0.11
	**n**	2.06	1.40	1.96	2.15	1.90	3.41	1.71	2.27	1.96	1.56	1.85	1.59
	**R^2**	0.95	0.96	0.91	0.89	0.87	0.92	0.95	0.98	0.95	0.97	0.97	0.98

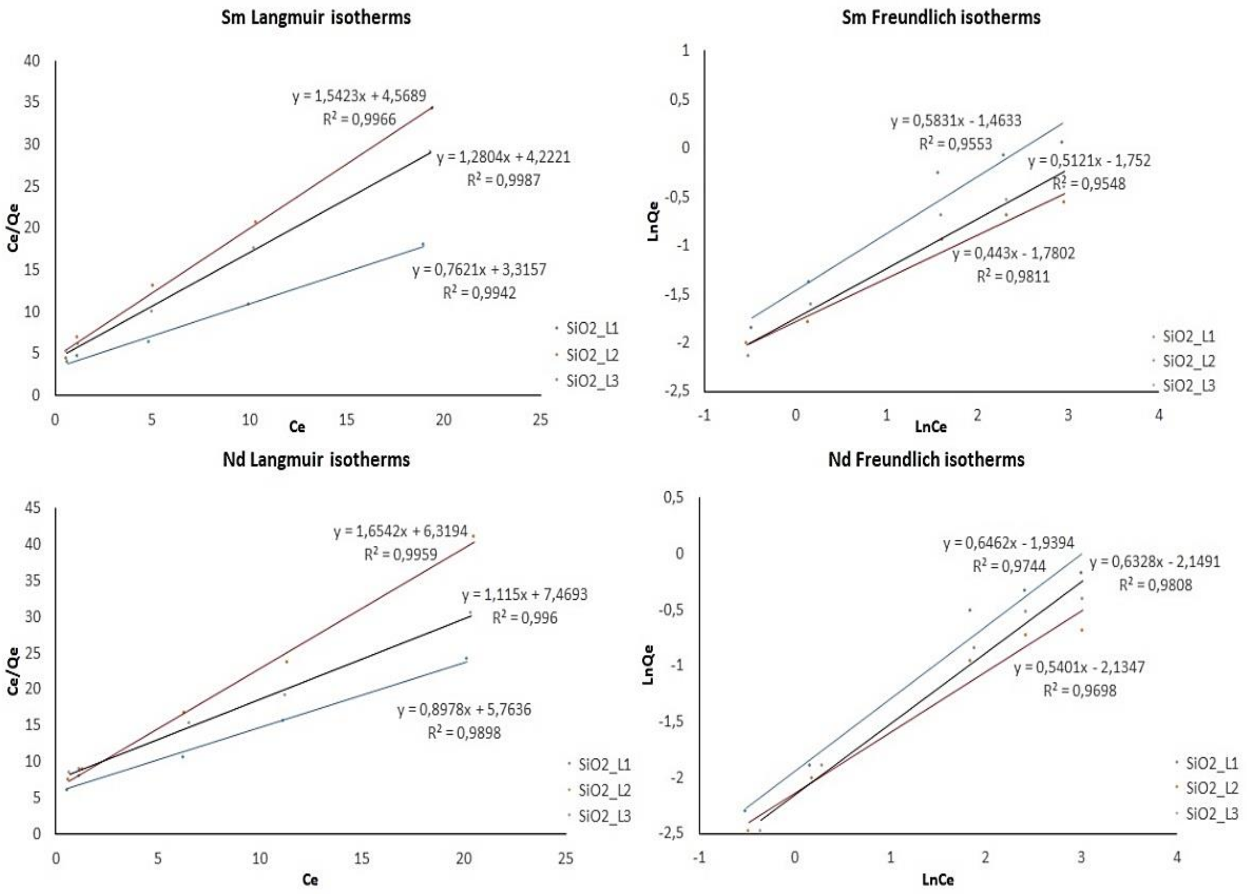

Figure S5. Linear plots of Langmuir and Freundlich isotherm models

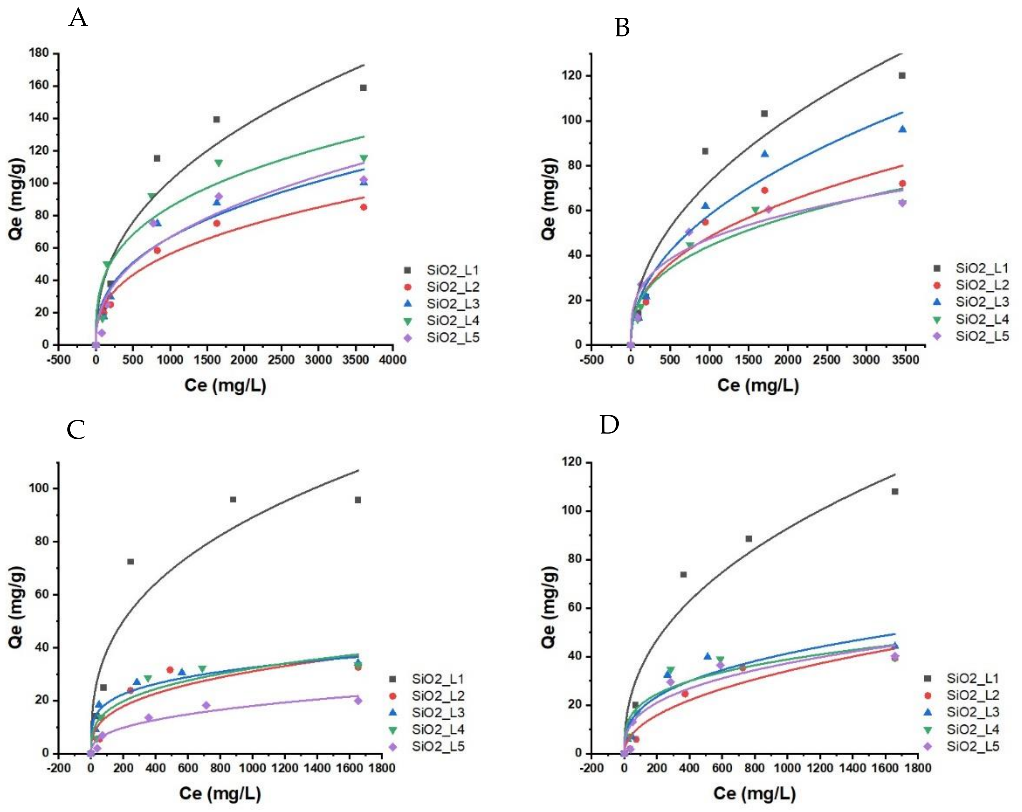

Figure S6. Freundlich adsorption isotherms of (**A**)-Sm, (**B**)-Nd, (**C**)-Ni and (**D**)-Co ions onto functionalized SiO_2 NPs

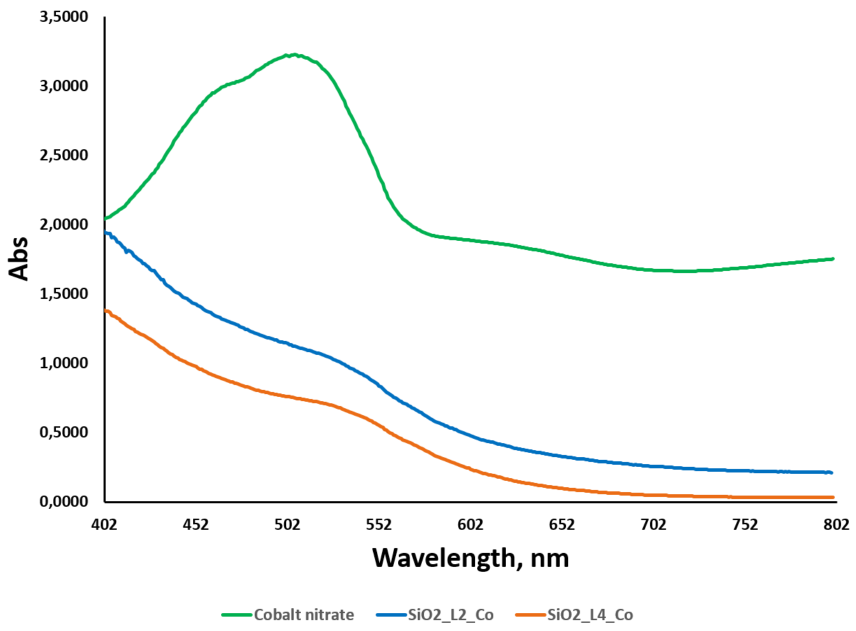

Figure S7. UV-Vis spectra of aqua complex of Co(II) and of adsorbents after uptake of cobalt(II) ions.

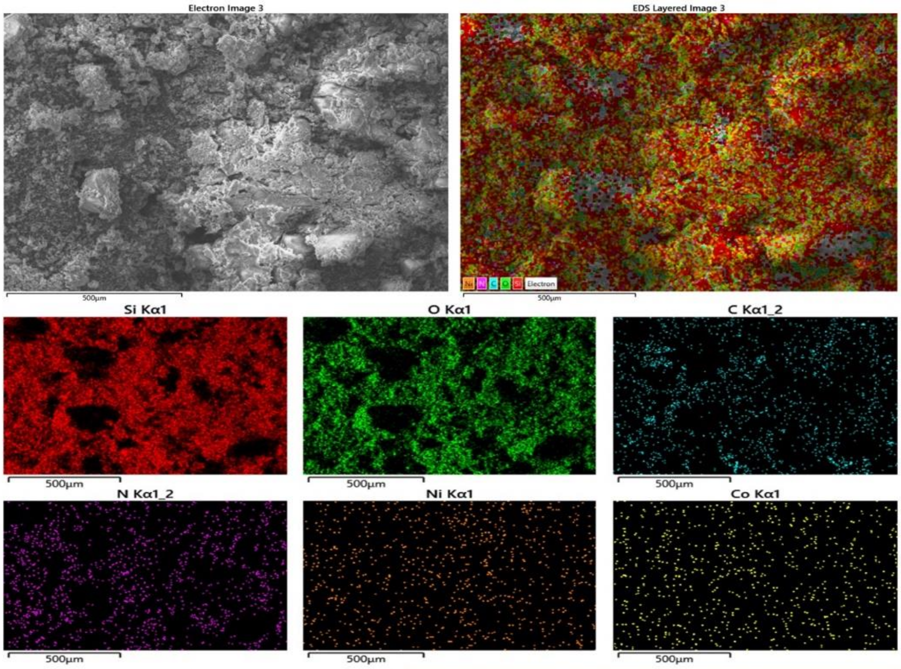

Map Sum Spectrum	
**Element**	**Atomic %**
**O**	58.81
**Si**	23.62
**Ni**	0.43
**C**	12.19
**N**	4.84
**Co**	0.12
**Total**	100.00

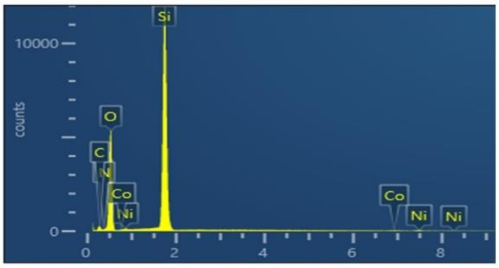

Figure S8. Example of EDS mapping of **SiO_2_L1** after adsorption of mixed Co + Sm metal ions EDS analysis and mapping data for the rest of the samples is available upon request.
